# Superimposed Intramammary Infection with *Staphylococcus hominis* Under Different Pre-Existing Infection Conditions in Bovine Udder: Bacterial Dynamics, Inflammatory Response and Oxidative Stress

**DOI:** 10.3390/ani16213497

**Published:** 2026-11-05

**Authors:** Wasana Chaisri, Duanghathai Saipinta, Montira Intanon, Noppason Pangprasit, Anyaphat Srithanasuwan, Witaya Suriyasathaporn

**Affiliations:** 1 School of Veterinary Medicine, Faculty of Veterinary Medicine, Chiang Mai University, Chiang Mai 50100, Thailand; wasana.ch@cmu.ac.th (W.C.); duanghathai.s@cmu.ac.th (D.S.); montira.intanon@cmu.ac.th (M.I.); 2 Research Center of Producing and Development of Products and Innovations for Animal Health and Production, Chiang Mai University, Chiang Mai 50100, Thailand; 3 Akkhraratchakumari Veterinary College, Walailak University, Nakhon Si Thammarat 80160, Thailand; p.noppason@gmail.com; 4 Department of Animal Sciences, Wageningen University, 6708 PB Wageningen, The Netherlands; numwan.sw@gmail.com; 5 Overseas Campus, Asian Satellite Campuses Institute, Nagoya University, Nagoya 464-8601, Japan

**Keywords:** bovine mastitis, co-infection, oxidative stress, *S. hominis*

## Abstract

Microbial interactions control mammary gland inflammation and disease severity. This study examined bacterial dynamics, inflammatory responses, and oxidative stress following *Staphylococcus hominis* (SH) superinfection in cows with chronic subclinical mastitis. Mammary quarters were assigned to varying SH doses: COW1 received 10^6^ and 10^5^ CFU/mL (two quarters each); COW2 received 10^6^ CFU/mL (two quarters) and PBS control (one quarter); COW3 received 10^4^ and 10^5^ CFU/mL (two quarters each); and COW4 received 10^4^ CFU/mL (two quarters). On Day 0, quarters received 5 mL of their assigned preparations. Milk and clinical parameters were evaluated during a pre-challenge baseline and monitored for 8 days post-inoculation. Results showed post-infection mastitis severity depended heavily on prior infections. SH alone functioned as a mild pathogen, inducing short-term inflammation with elevated somatic cell counts and IL-6 levels primarily on Days 1 to 3, but did not independently cause severe tissue damage. The intense immune response, elevated malondialdehyde (MDA) levels, and severe tissue damage were primarily driven by pre-existing major mastitis pathogens. Although *S. hominis* is inherently a mild pathogen, high bacterial loads synergistically exacerbate oxidative stress and clinical mastitis symptoms in co-infected quarters.

## Simple Summary

This study investigates how the mammary microbiota, specifically *Staphylococcus hominis*, behaves under different pre-existing infection conditions. The severity of mastitis and tissue damage was largely influenced by bacteria that were already present in the udder. High levels of *S. hominis* may, however, enhance inflammation and oxidative stress in quarters already infected with major mastitis pathogens. These findings highlight the importance of interactions between bacterial species in determining mastitis severity.

## 1. Introduction

Superimposed infection refers to a new infection that occurs on top of an existing infection. For example, a bacterial infection that develops after a viral infection, such as influenza, is a common type of superinfection. This can complicate the clinical course, making diagnosis and treatment more difficult. In human medicine, superimposed infections are critical because they often worsen patient outcomes, especially in vulnerable populations such as children, the elderly, and immunocompromised patients [1,2]. However, in veterinary medicine, the concept of superimposed infection has been explored from a different perspective through the lens of bacterial interference and competitive exclusion. Introducing mild or commensal bacteria could theoretically prime the local innate immune response or outcompete major pathogens for resources. While preliminary proof-of-principle studies in mouse models [3,4] have shown that mild superinfections can trigger immune responses leading to the clearance of *Staphylococcus aureus*, the direct application and efficacy of this dynamic in chronic bovine mastitis remain largely unexplored.

Bovine mastitis is mainly associated with multiple microbial infections, especially in high-prevalence mastitis herds [5,6]. Chronic mastitis represents a distinct intramammary environment in which persistent intramammary infection establishes a “chronic infection niche” characterized by altered local immune responses, tissue damage, and disruption of the mammary microbial ecosystem [7]. These changes facilitate colonization by additional microorganisms and contribute to superimposed intramammary infections [8]. Non-aureus staphylococci (NAS), historically considered minor pathogens, are mostly diagnosed as concomitant with either major or minor pathogens. The pathogenic potential of non-aureus staphylococci (NAS) in the bovine mammary gland was experimentally established in the early 1970s [9], with comparative studies demonstrating that staphylococcal virulence is species-dependent; specifically, *S. aureus* exhibits significantly higher invasiveness and induces more severe histopathological changes than NAS species [10]. Building upon these foundational works, subsequent research utilizing advanced species-level identification has confirmed that NAS comprise a highly heterogeneous group characterized by distinct, species-specific variations in virulence and host response. The most frequently isolated NAS are *Staphylococcus. chromogenes*, *Staphylococcus haemolyticus*, *Staphylococcus simulans*, *Staphylococcus xylosus*, *Staphylococcus epidermidis*, and *Staphylococcus hyicus* [11,12]. Previously, a specific strain of *S. hominis* has been proposed as a potentially beneficial organism due to its inhibitory activity against major mastitis pathogens such as *S. aureus* and *Streptococcus agalactiae* in both in vitro and in vivo settings by suppressing the proliferation or survival of these pathogens [4,13], decreasing inflammation, and reducing damage in mammary tissue in mice [3,14]. The interactions among bacteria within the mammary gland could influence inflammation, bacterial load, and disease outcomes. Immune modulation, including altered neutrophil recruitment and changes in cytokine profiles, contributed to complete bacterial clearance, resulting in spontaneous recovery [15]. Despite extensive research on mastitis pathogens and host immune responses, important gaps remain in understanding superimposed intramammary infection (IMI) under different pre-existing infection conditions in bovine udder. Although certain strains of *S. hominis* exhibit in vitro inhibitory effects against major mastitis pathogens, there is limited evidence regarding their behavior in bovine udder under pre-existing infection conditions. In such environments, ongoing inflammation may alter bacterial competition, coexistence, and host responses, potentially affecting whether disease outcomes are reduced or progress to more severe clinical states.

However, the potential of a specific strain of *S. hominis* bacteria proven beneficial against experimental IMI in mice [3] has not been explored for mastitis quarters in dairy cattle. The objective of this study was to evaluate the bacterial dynamics, inflammatory response (somatic cell and cytokine response), and cell damage by oxidative stress response associated with *S. hominis* superinfection in bovine quarters with different pre-existing infection conditions. Understanding these interactions may contribute to a better understanding of host–pathogen dynamics during superimposed intramammary infection and provide a basis for further investigation of biologically based approaches to mastitis management.

## 2. Materials and Methods

### 2.1. A Specific Strain of S. hominis and Its Preparation

A strain of *Staphylococcus hominis* (SH) used in this study has been proven to have beneficial activity against major mastitis pathogens in our previous studies, either in vitro studies with standard culture media or in milk [16], or in vivo studies using mice at different bacterial concentrations [3]. The strain of SH was recovered from a glycerol stock stored at −80 °C in Tryptic Soy Broth (TSB) and incubated at 37 °C for 16–18 h. For a single colony, the bacterial suspension was then streaked onto tryptic soy agar (TSA) plates. The bacterial suspension was adjusted to a 0.5 McFarland turbidity (~1 × 10^8^ CFU/mL) and serially diluted in sterile phosphate-buffered saline (PBS). Three concentrations of fresh bacterial suspensions were prepared for intramammary inoculation. Prior to inoculation, the bacterial concentration in the mammary gland was adjusted to 10^6^, 10^5^, and 10^4^ CFU/mL. To verify the exact viability and purity of the delivered inoculum, an aliquot from each final suspension tube (including the sterile PBS control) was collected immediately prior to intramammary administration. These aliquots were subjected to drop plating (50 µL) onto TSA plates and incubated at 37 °C for 24 h. Subsequent colony enumeration confirmed the actual delivered doses of 10^6^, 10^5^, and 10^4^ CFU/mL, while colony morphological evaluation ensured the absolute purity of the *S. hominis* suspension and the sterility of the PBS control. The suspension was aliquoted into three sterile 15 mL centrifuge tubes (10 mL per tube) and kept on ice until administration. The 0 CFU/mL control was prepared using sterile, pyrogen-free PBS.

### 2.2. Animals and Experimental Design

The Institutional Animal Care and Use Committee at Chiang Mai University’s Faculty of Veterinary Medicine (permission number R19/2565) in Thailand approved all animal care and sample collections. This study was conducted between November 2023 and February 2024, using 4 dairy cows with a history of chronic and recurrent subclinical mastitis from 2 smallholder dairy farms in Chiang Mai Province, Thailand. The cows used were on the culling plan during their drying-off period or within the next few months. The cause of culling was due to their chronic subclinical mastitis after treatment with antibiotics at least 3 times and re-infection within the same lactation. These cows also had several experiences with clinical mastitis. All cows were purchased before the start of the experiment. The experiment on the first two cows (COW1 and COW2) was conducted at the smallholder dairy farms where both cows were kept. The experiment on the other two cows (COW3 and COW4) was conducted in the training university barn at the Faculty of Veterinary Medicine, Chiang Mai University, Thailand. After purchase, the cows were monitored for health and cared for at the experimental sites for at least 1 week, in accordance with standard procedures. Briefly, cows were milked twice a day with a budget-type milking machine, fed roughage ad libitum, supplemented with concentrates according to their milk production, and raised in the area where cleaning was performed twice a day. All systemic clinical signs of disease, as indicated by systemic pathological conditions such as lethargy, reduced appetite, hyperthermia, cardiac arrhythmias, or diminished ruminal function, were treated, and animals were allowed 1 week to recover before starting the experiment. Predefined endpoints of the study included more severe systemic illness, such as recumbency or complete inability to eat or drink.

An exploratory, quarter-level intramammary challenge experiment was conducted using a partially split-udder configuration. The 14 functional mammary quarters were allocated to four *S. hominis* concentration groups (0, 10^4^, 10^5^, and 10^6^ CFU/mL). Because of the exploratory nature and limited number of available cows and functional quarters, the allocation was not balanced within each cow, and not all cows received all four concentrations or a PBS control. On day 0, after morning milking, each cow’s mammary quarters were challenged with 5 mL of each of the four different suspensions using a 16G × 2″ sterile catheter (Nipro, Bangkok, Thailand). Based on the random allocation, COW1 was challenged with 10^6^ CFU/mL for the left-front (LF) and right-front (RF), or quarters 1 and 2, respectively, and 10^5^ CFU/mL for the left-hind (LH) and right-hind (RH), or quarters 3 and 4, respectively. Two mammary quarters from COW2 were infected with *S. hominis* at a dose of 10^6^, whereas the other quarter was administered phosphate-buffered saline. Two mammary quarters from COW3 were infected with *S. hominis* at a concentration of 10^4^, whilst the remaining two udders were infected with *S. hominis* at a concentration of 10^5^. Two full quarters of COW4 were contaminated with *S. hominis* at a concentration of 10^4^, whereas one quarter was nonproductive, as illustrated in Table 1.

**Figure 1:**
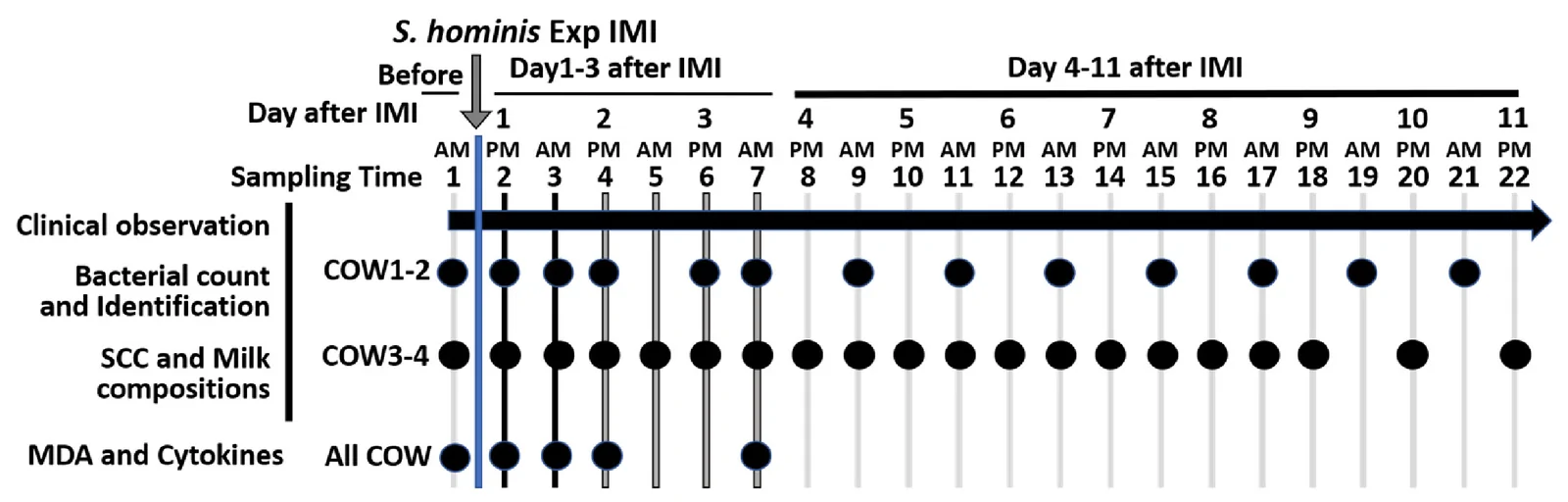
Experimental design.

After inoculation, the data collection and sampling schedule are shown in Figure 1. During the study, clinical examination and health monitoring of COW1 and COW2 were conducted by the farmers and local veterinarians, while those of COW3 and COW4 were conducted by veterinary students and university staff. Mastitis was separated into 3 grades: Mild: abnormal milk with flakes, clots, or discoloration; Moderate: abnormal quarter with painful swelling, redness, and a feeling of warmth; and severe: systemic signs such as fever and anorexia [17]. Due to differences among experimental sites, the schedule for aseptic milk sample collection for bacterial counts, identification, and milk composition measurements varied. For the first 7 sampling times, except for time 5, milk samples were collected twice daily from all cows at 12 h intervals (morning and afternoon). From sampling days 4 to 11 after experimental IMI, milk from COW1 and COW2 was collected once daily at 24 h intervals, whereas milk samples from COW3 and COW4 were collected twice at 12 h intervals from sampling times 1 to 18, and once a day after that. At each sampling, cows were clinically examined for rectal temperature, udder abnormalities, and milk appearance. Milk samples were refrigerated and divided into two aliquots. One was immediately used for bacteriological culture and identification, while the other was stored at −20 °C for further evaluation of milk composition, somatic cell count (SCC), cytokines, and malondialdehyde (MDA).

### 2.3. Sample Collection

Milk samples were collected, including both sterile and non-sterile milk. Prior to collecting a sterile milk sample, teats were thoroughly cleaned with 70% ethanol, and the first few streams of milk were discarded to reduce contamination. Approximately 3–5 mL of milk was aseptically collected into sterile bottles for counting and identification. Non-sterile milk samples (45–50 mL) were collected in a sterile centrifuge tube. These samples were separated into two groups: 30 mL for milk composition and somatic cell count (SCC) measurement, and 15 mL for cytokine and malondialdehyde (MDA) analysis. All milk samples were immediately transferred on ice to the laboratory for further analysis. The milk SCC and compositional parameters were measured using Fossomatic^TM^ FC (Foss, Denmark).

### 2.4. Bacterial Enumeration and Identification

The drop plate technique was used for bacterial enumeration. Five types of solid media, including Plate Count Agar (PCA), Baird–Parker agar (BPA) for presumptive *Staphylococcus* spp., Edwards medium agar for presumptive *Streptococcus* spp., and MacConkey agar for presumptive Gram-negative organisms, were used. Each milk sample was serially diluted 10-fold, and 10 µL was transferred to each solid medium, which was then incubated at 37 °C for 16–18 h. Only plates containing 3–30 colonies were considered for enumeration, and bacterial counts were expressed as colony-forming units per milliliter (CFU/mL). Bacteriological identification was carried out in accordance with NMC recommendations [18]. Colonies with distinctive morphologies were picked from the agar plates and sub-cultured on 5% blood agar until pure isolates were produced. Purified bacterial isolates were delivered to the Office of Scientific Instrument and Testing at Prince of Songkhla University in Songkhla, Thailand, for species identification via matrix-assisted laser desorption/ionization time-of-flight mass spectrometry (MALDI-TOF MS) using the direct transfer protocol. Identification was performed according to the manufacturer’s instructions using intact ribosomal protein profiles. Spectral data were analyzed with Bruker Biotype and Real-Time Classification 3.0 software. The analysis included only isolates with a species-level identification score ≥2.00 (log score ≥2.00), as specified by the manufacturer.

### 2.5. Cytokine Quantification

Milk samples stored at −20 °C were thawed and centrifuged at 10,000× *g* for 20 min at 4 °C. After centrifugation, the top fat layer was discarded and the defatted cell-free supernatant was carefully collected, transferred to clean tubes, and stored at −20 °C for a duration of 4 weeks until further analysis for cytokine quantification. IL-4 and IL-6 concentrations were quantified using a commercial ELISA kit for bovine IL-4 and IL-6 (Immunology Consultants Laboratory, Portland, OR, USA) according to the manufacturer’s protocol. Briefly, 100 µL of standards, samples, and controls were added to ELISA plate wells and incubated for 30 min at 37 °C for IL-4 or for 1 h at room temperature with gentle shaking. Following aspiration and 3 to 4 washes with wash buffer, 100 µL of antibody solution was added and incubated for 1 h at 37 °C for IL-4 and for 30 min at room temperature in the dark for IL-6. After washing, 100 µL of streptavidin–HRP solution was added, and the mixture was incubated for 30 min at 37 °C in the dark. Following the final washes, 100 µL of Tetramethylbenzidine (TMB) substrate solution was added, and the mixture was incubated for 15 min in the dark. The reaction was terminated with 100 µL of stop solution, and optical density was measured at 450 nm within 30 min using a microplate reader. The detection ranges of IL-4 and IL-6 for cattle were 3.906–500 pg/mL and 3.91–1000 pg/mL, respectively, as reported by the manufacturers.

### 2.6. Malondialdehyde (MDA) Measurement

The concentration of malondialdehyde in milk was assessed utilizing high-performance liquid chromatography (HPLC) as described by Fenaille et al. [19]. Add 800 µL of TBA/TCA reagent (1% thiobarbituric acid/5% trichloroacetic acid) and 800 µL of Butylated Hydroxytoluene (BHT) (0.8%) to 1000 µL of milk. Calibration standards were prepared by replacing milk with 1000 µL of 1,1,3,3-tetraethoxypropane (TEP) solutions at concentrations of 1.875, 0.938, 0.469, 0.234, and 0.117 nmol/mL. The mixture was centrifuged at 2700× *g* for 10 min. The supernatant was heated to 70 °C for 1 h, then cooled and filtered through a 0.2 µm membrane before HPLC analysis. MDA concentrations were determined using a Shimadzu Corporation HPLC system (Kyoto, Japan) equipped with an LC-10AD pump, SPD-10A UV–Vis detector, and CBM-20A system controller. Chromatographic separation was done with an Inertsil^®^ ODS-3 C18 column (4.6 × 250 mm, 5 µm; GL Sciences, Tokyo, Japan). The mobile phase comprised a 50 mM phosphate buffer (pH 6.0) prepared by dissolving 5.865 g KH_2_PO_4_ (potassium dihydrogen phosphate; MW 136.09) and 1.203 g K_2_HPO_4_ (dipotassium hydrogen phosphate; MW 174.18) in 600 mL of water and diluting to 1000 mL (A) and methanol (B). The separation occurred under isocratic conditions, with an A: B ratio of 60:40 (*v*/*v*). The flow rate was 1.0 mL/min, and the injection volume was 20 µL. The column temperature was maintained at 35 °C, and detection was performed at 532 nm using a UV detector. The run time was fifteen minutes.

### 2.7. Statistical Analysis

Prior to the final statistical analysis, all continuous data were tested for normality. Non-normally distributed data were subjected to natural-logarithm transformations. Percentages were used to describe categorical variables. The outcomes evaluated the effects of superimposed IMI with *S. hominis*, including categorical variables: percentages of body temperatures and clinical mastitis, and continuous variables: maximum bacterial counts, somatic cell counts (SCC), and MDA, IL4, and IL6 levels. For SCC, based on the performance range (1–9999 × 10^3^ cells/mL) of our instrument, SCC data exceeding 9999 × 10^3^ cells/mL, or milk with clinical mastitis, were set to 9999 × 10^3^ cells/mL. Multiple logistic regression (PROC GENMOD, SAS OnDemand for Academics version 9.4) was repeated to analyze the categorical variables, while linear mixed regression analysis (PROC MIXED, SAS OnDemand for Academics) was used to model the dependent continuous variables. All models included periods of SH-IMI (Before (D0), D1–3, and D4–10), Cow (1–4), SH-IMI concentrations (0, 10^4^, 10^5^, and 10^6^), periods of antipyretic use (0,1), and periods of antibiotic use (0, 1). Quarters within cows were treated as random effects, and an autoregressive type 1 correlation structure was specified. Least-squares means of SH-IMI period, each cow, and SH-IMI concentration were used to describe the results from the final analysis. The significance level was set at *p* < 0.05.

## 3. Results

**Figure 2:**
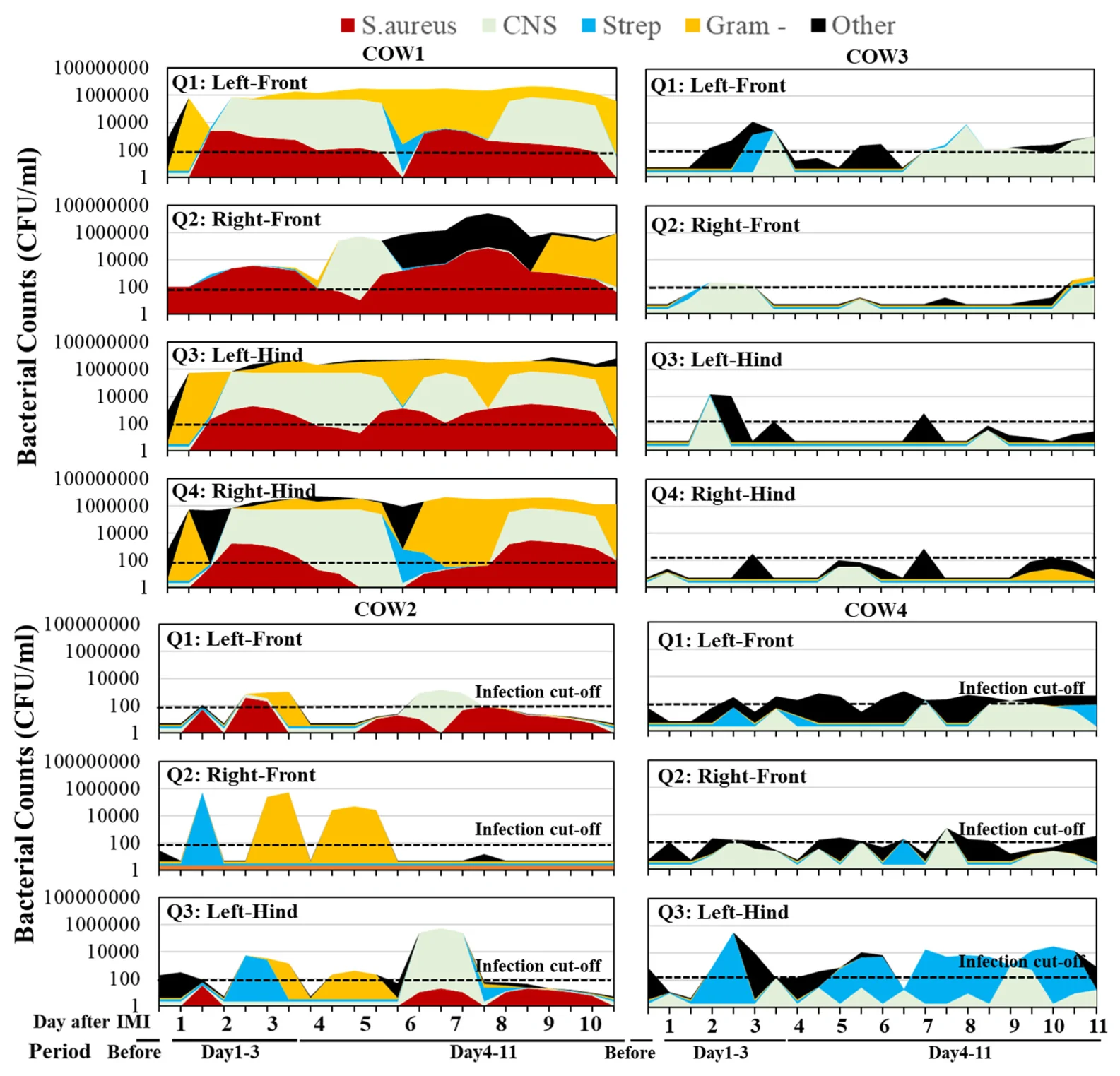
The figure depicts quarter-level bacterial community dynamics (CFU/mL; log scale) for each cow and quarter: left-front (Q1), right-front (Q2), left-hind (Q3), right-hind (Q4). Stacked areas represent bacterial groups (red = *S. aureus*, light green = coagulase-negative staphylococci [CNS], blue = streptococci, yellow = Gram-negative, black = other), and the dashed horizontal line denotes the infection cut-off.

Overall conditions of all cows during the study are shown in Table 1. As detailed in Table 1, a total of 14 quarters were evaluated across the four cows; among them, the right quarters of COW2 and COW4 were identified as non-producing (NPU) units. These quarters were pre-existing non-functional (blind) quarters prior to the commencement of the study, yielding no milk samples, and were therefore excluded from the longitudinal milk parameter collections. Before experimental IMI, COW1 was infected with *Staphylococcus aureus* in all quarters, while two quarters of COW4 were infected with *Streptococcus agalactiae*, and one quarter of COW3 was infected with *Streptococcus uberis*. After superinfection with *S. hominis* at concentrations of 10^5^ and 10^6^ in udders originally infected with *S. aureus* (COW1), *S. uberis* (COW2), and *S. agalactiae* (COW4), *S. hominis* was undetectable 1 day post-infection. Conversely, *S. hominis* was identified exclusively in COW3 at a very low concentration (Figure 2), with no bacteria being detected prior to the experiment. High percentages of clinical mastitis quarters were found for COW1 (75%), COW2 (66.7%), and COW4 (66.7%), but only 25% (1/4) was found for COW3. Consequently, fever occurred in COW1, COW2, and COW4. Most of the originally infected bacteria remained at day 1. Contamination with *S. agalactiae* was detected in the left-front quarter of COW3, but it resolved by day 10. Infections with *S. aureus* and *S. agalactiae* persisted and were still detected on day 10, but *S. uberis* in COW2 was not detected.

**Figure 3:**
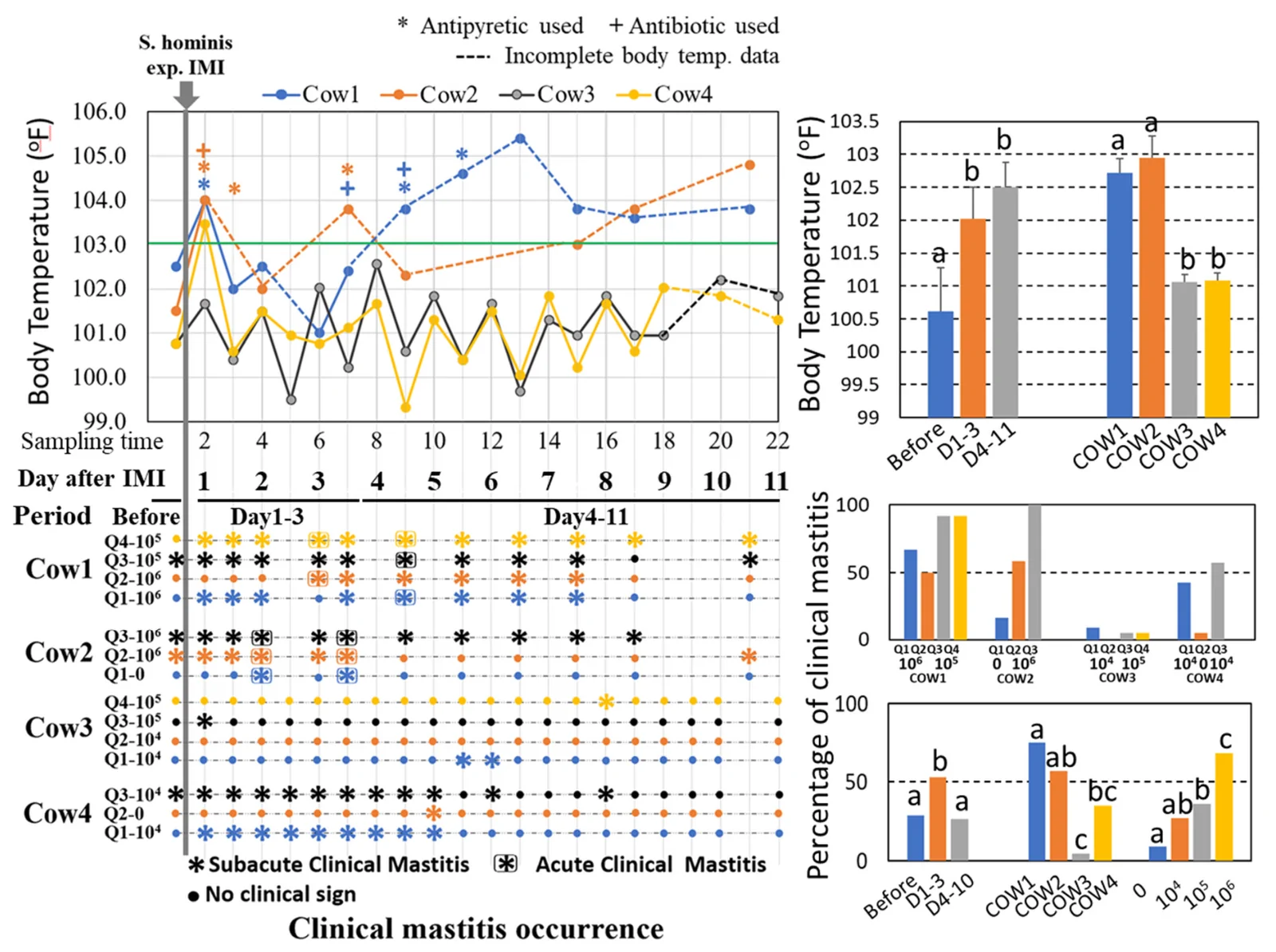
Body temperature and clinical mastitis signs during the IMI experiment and natural observation after experimental IMI. a, b, c: different letters indicate significant differences at *p* < 0.05.

The body temperatures and clinical appearance of all cows and quarters, recorded as mild and moderate, are presented in Figure 3. After being infected with *S*. *hominis*, all cows’ body temperatures increased, and the temperatures of three cows primarily infected with other bacteria exceeded the fever threshold of 39.7 °C [20]. COW1 and COW2 expressed severe clinical mastitis; local veterinarians prescribed antipyretics (periods 2 and 3) and antibiotics (period 2) for COW2, and only antipyretics for COW1. During periods 7 to 9, antibiotics or antipyretics were sometimes administered to COW1 and COW2 when their body temperatures exceeded the fever threshold. At day 10 (periods 21–22), COW1 and COW2 remained febrile. In contrast, COW3 and COW4 had normal temperatures throughout the study period. In terms of clinical mastitis appearance, most cases were mild, except for periods in certain quarters of COW1 and COW2, which showed moderate clinical mastitis. Four quarters, including Q3 of COW1, Q2 and Q3 of COW2, and Q3 of COW4, had abnormal milk and mild clinical mastitis before inoculation with *S*. *hominis*. During the first week after experimental IMI, 4 quarters of COW1, Q3 of COW2, and Q1 and Q3 of COW4 showed mostly clinical signs of mastitis. The body temperature of all cows significantly increased after being inoculated with *S*. *hominis* compared to before, but there was no effect of antipyretic and antibiotic administration. The incidence of clinical mastitis increased with bacterial concentration, with a higher percentage at higher concentrations than at lower concentrations. COW1 and COW2 had a higher risk of clinical mastitis than COW3 and COW4.

Bacterial loading after the experimental IMI, separated into their genus, is shown in Figure 2. The experimental *S*. *hominis* infection subsequently disappeared in almost all quarters. In the most severe mastitis, COW1 had superinfection, infection with more than 1 bacterium, in all quarters, primarily due to *S*. *aureus*, with concentrations exceeding the infection cut-off of 100 CFU/mL. Interestingly, superinfection with Gram-negative bacteria in those quarters caused clinical mastitis. In COW2, superinfection occurred at fewer severity levels, as indicated by the bacterial count. Quarter 3 (left-hind), originally with *S*. *uberis* infection and clinical mastitis for several days, showed an increase in the low bacterial count of *S*. *aureus* several times. COW3, with the lowest clinical mastitis quarter, shows mostly infection by the minor pathogen, CNS, with a few times when the counts exceeded the cut-off levels. COW4 was infected by *S*. *agalactiae* in Q1, and Q3 shows an increase in some Streptococci above the cutoff, especially in Q1. 

**Figure 4:**
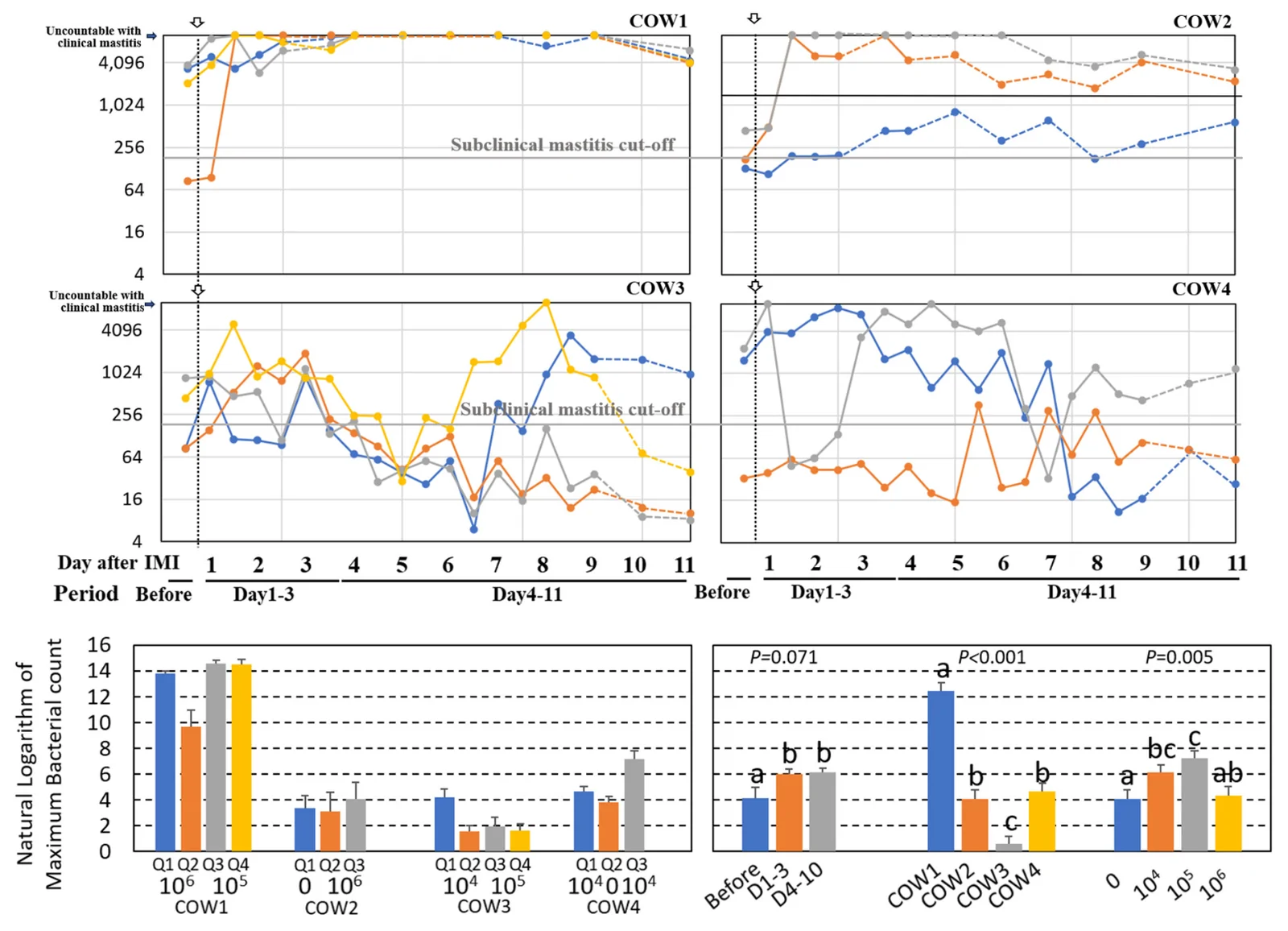
Maximal bacterial counts, separated into quarters across collecting periods (above). Averages and standard errors of the means for the natural logarithm of the maximal bacterial count at the quarter levels are presented at the bottom left. Least-squares means and standard errors of the means for all bacterial counts across all levels of period of SH-IMI, cow factors, and SH-IMI concentration from a generalized linear mixed model are shown below. a, b, c: different letters indicate significant differences at *p* < 0.05.

Maximal bacterial counts, separated into quarters across collecting periods, are shown in Figure 4 (above). COW1 consistently had counts above the infection cut-off, whereas COW2, COW3, and COW4 had counts below the cut-off at several collection periods. Averages of the natural logarithm of maximal bacterial count are shown in Figure 4 (left-below). At the quarter level, the bacterial count across all quarters of COW1 was higher than across all quarters of the other three cows. Results from the final model of the bacterial count show that cow and SH-IMI concentration groups were significantly related to bacterial counts, while the effects of IMI timing were tentatively different (*p* = 0.07), with no effect of antipyretic and antibiotic administration. Across levels, the bacterial count increased significantly after SH-IMI. COW1 was significantly higher than COW2 and COW4 (*p* < 0.05). Interestingly, the bacterial count after correcting for the COW and time factors showed that 10^5^ was the highest and significantly higher than 0 and 10^6^.

**Figure 5:**
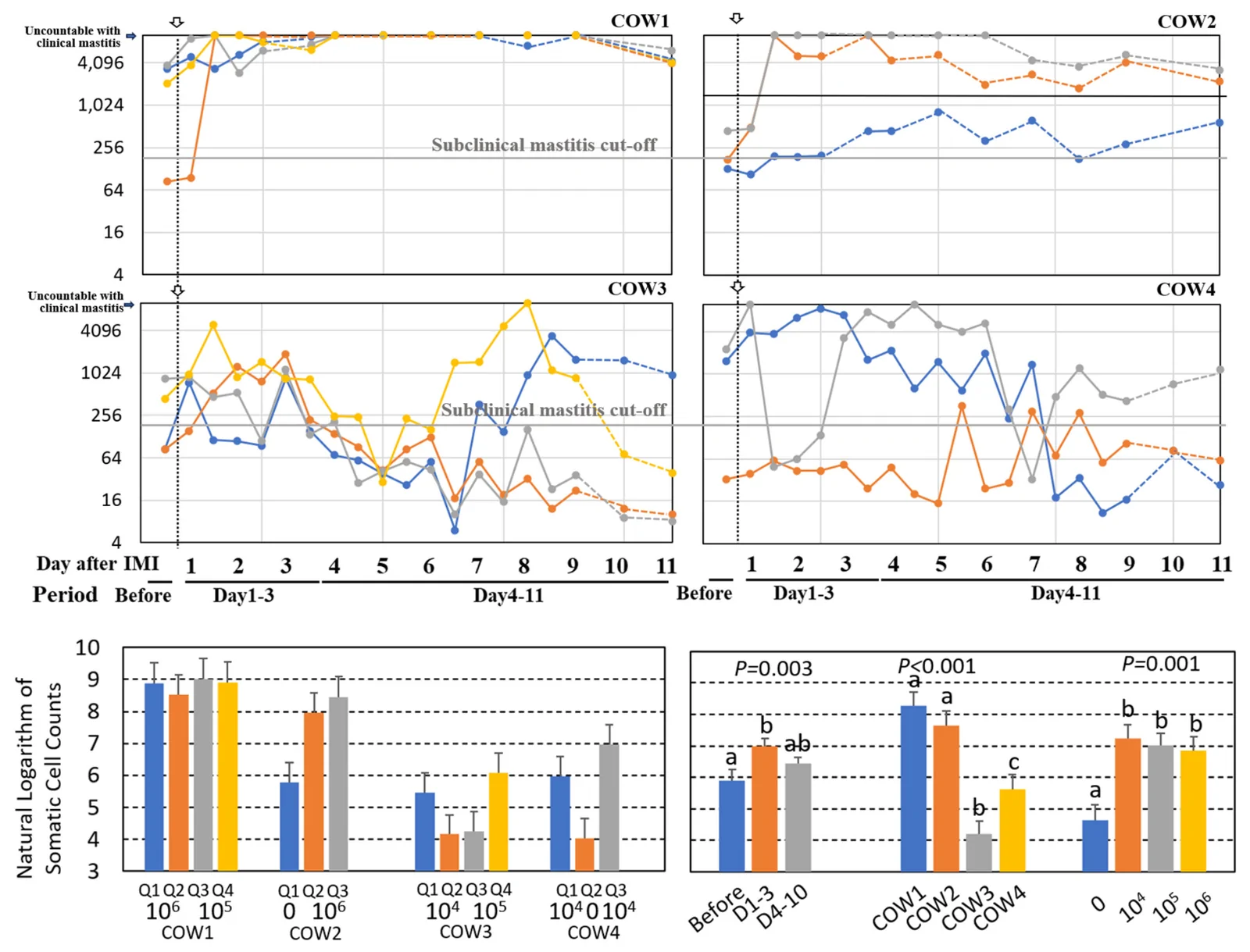
Quarter somatic cell count (SCC during experimental IMI with *S. hominis* (SH-IMI) at different concentrations, separated for each cow, is shown (above). Means and standard errors of the natural logarithm of SCC are presented at the bottom left. Least-squares means and standard errors of the means for all SCC at all levels of period of SH-IMI, cow factors, and SH-IMI concentration from a multiple generalized linear mixed model are shown below on the right. a, b, c: different letters indicate significant differences at *p* < 0.05.

Regardless of clinical mastitis, all milk quarters were submitted for SCC measurement, and the results were based on the machine’s SCC, as shown in Figure 4. With the cutoff at 200 × 10^3^ cells/mL for subclinical mastitis, all times across all quarters of COW1 were defined as mastitis, either subclinical or clinical. Regarding SCC level, both Q2 and Q3 of COW2 were mastitis, subclinical or clinical, at all times. COW3 showed increases in SCC at subclinical mastitis levels, but only 2 quarters remained subclinical mastitis in the second half of the study. Except for one sample, all COW4 samples were originally infected with *S. agalactiae* in Q1 and Q3, resulting in clinical mastitis, but SCC data could not be retrieved from the clinical mastitis milk samples. Q2 of COW4 had SCC below the subclinical mastitis level for most of the study period. After setting the SCC at 9999 × 10^3^ cells/mL for undetectable SCC of clinical mastitis to be able to calculate means and standard errors of means, the means and standard errors of the means of the natural logarithm of SCC across days after SH-IMI are shown in Figure 5 (above). Averages of SCC of all quarters from COW1 and Q2 and Q3 of COW2 were high, while all quarters from COW3 and COW4 were low (Figure 5, below-left). The final model indicates that most variables, excepting antipyretic and antibiotic administration, were significantly related to SCC levels (Figure 5, below-right). The average SCC on days 1–3 increased significantly after SH-IMI, then decreased on days 4–10 (*p* < 0.01). The SCC was lowest in COW3, and the highest in COW1 and COW2, which were significantly higher than in COW3 and COW4. Inoculated with PBS (Group 0) was significantly lower than the other three groups.

**Figure 6:**
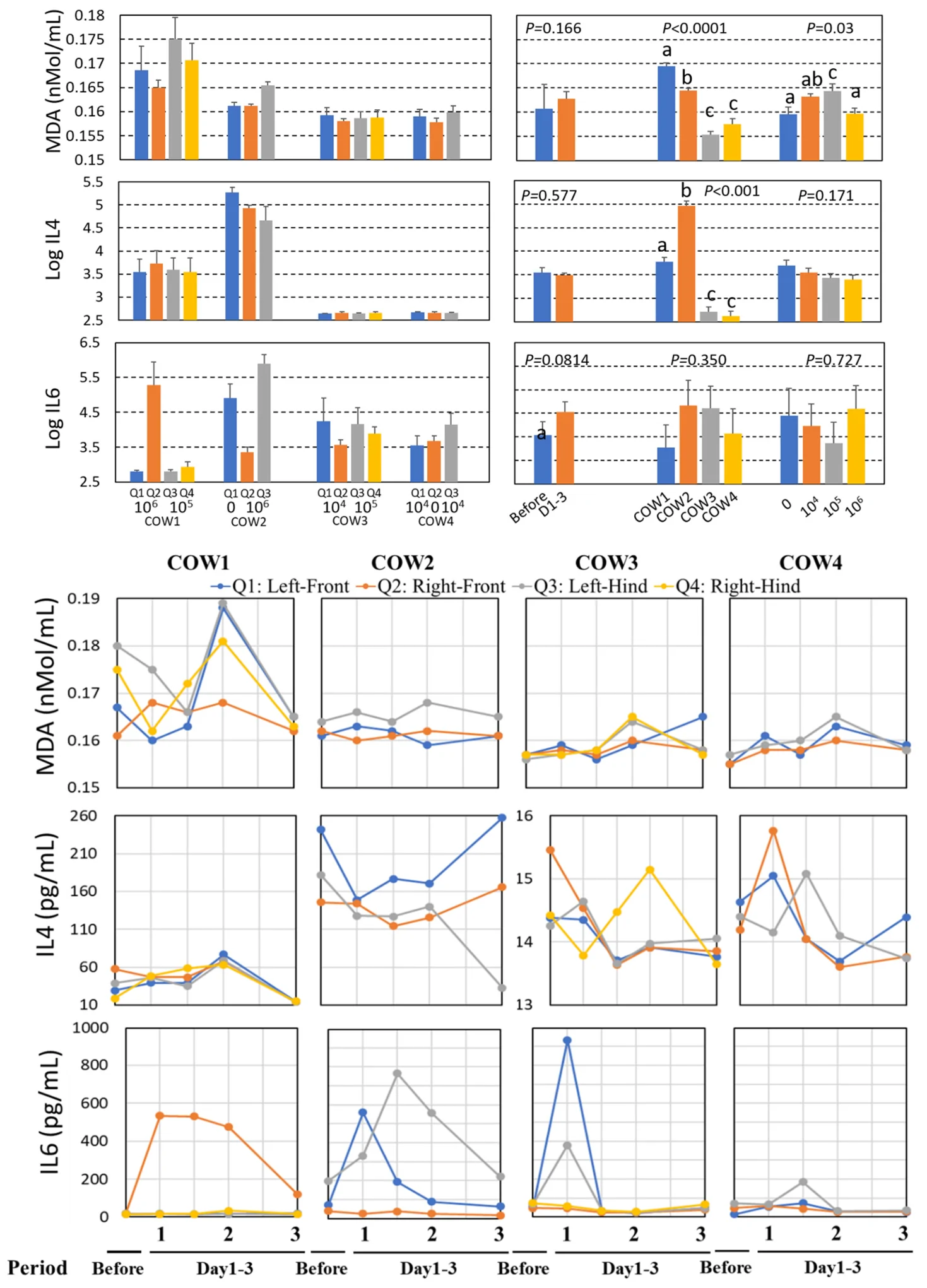
Concentrations of quarter MDA, logarithms of IL4 and IL6 across sampling times separated into cows are shown (above). Means and standard errors of the means (SEMs) for all substances are presented at the bottom left. Least-squares means and SEMs for all substances, separated by SH-IMI period, cow factors, and SH-IMI concentration, from a multiple generalized linear mixed model are shown below on the right. a, b, c: different letters indicate significant differences at *p* < 0.05.

Concentrations of malondialdehyde (MDA), IL-4, and IL-6 at the first 3 days after the experimental SH-IMI were shown in Figure 6. The MDA levels in COW1 with superinfection, higher bacterial count, higher SCC, and *S. aureus* were higher than those in the other cows, indicating greater severity in COW1. For COW2, Q3 (left-hind) had higher MDA than the other quarters of the same cow, whereas no differences were observed for COW3 and COW4. The high levels of IL-4 were found in COW2, followed by COW1, ranging from 14 to 258 pg/mL, while IL-4 in COW3 and COW4 was very low, ranging from 13.6 to 15.8 pg/mL. For the first time, IL-4 in COW2 across all quarters was already higher than 145 pg/mL and remained above 110 pg/mL for at least 3 subsequent periods. In quarter 2 (right-front) of COW1, the absence of clinical signs during the early period was concomitant with higher IL-6 levels. Spikes in IL-6, especially immediately after experimental IMI, were also observed in Q1 and Q3 of COW2 and COW3, respectively. All spikes occurred between 24 and 36 h after experimental IMI.

Averages of MDA, IL-4, and IL-6, separated by quarter for each cow, are shown in Figure 6 (bottom-right). The MDA averages for the quarters from COW1 were obviously higher than those for the others. Interestingly, the average IL-4 across all quarters from each cow was similar, with IL-4 from COW2 the highest, followed by COW1, COW4, and COW3. In contrast, IL-6 levels differed between quarters in the same cows. The lowest IL-6 levels were observed in COW1 at Q1, Q2, and Q4. Least-squares means for MDA, IL-4, and IL-6 from the final models are shown in Figure 6 below. There was no significant effect of antipyretic or antibiotic administration on MDA, IL-4, or IL-6. The cow factor and SH-IMI concentrations were significantly related to MDA. MDA of COW1 was significantly higher, whereas those of COW3 and COW4 were significantly lower than those of COW1 and COW2. Inconsistency in the SH-IMI concentration was found. SH-IMI at 10^5^ was higher than the others, even for 10^6^ (*p* < 0.05). The cow factor was the only factor related to IL-4 concentrations. No significant factor was found for IL-6.

## 4. Discussion

The results of this study reveal that the severity of mastitis following superimposed intramammary infection with *S. hominis* (SH-IMI) varies significantly, influenced by cow-specific factors and prior persistent infections. The response following SH-IMI indicates short-term inflammation, particularly in D1 to D3, with elevated SCC and IL-6 levels, potentially associated with bacterial load in the udder. The colonization of *S. hominis* was not observed in environments with pre-existing infections from major pathogens such as *S. aureus*, *S. agalactiae*, and *S. uberis*. *S. hominis* has been proven as a particular strain beneficial for the in vitro suppression of major mastitis pathogens [21]. Nevertheless, our field study found the absence of *S. hominis* one day post-superinfection in almost every quarter exhibiting these prior infections. In contrast, *S. hominis* was detected at a low level in COW3, indicating the absence of any preexisting organisms prior to superinfection. This pattern supports the concept that chronically infected quarters may represent an already-occupied “niche,” both microbiologically and immunologically, hindering the establishment of newly introduced organisms or leading to the fast clearance of *S. hominis* [22,23]. In addition, pre-existing infection of a major pathogen can occupy adhesion sites, utilize nutritional niches, and maintain an inflammatory state that elevates SCC and primes innate defenses, limiting the ability of *S. hominis* to persist [11]. Consistent with the findings of De Vliegher et al. [5] and Adkins & Middleton [24], the colonization time of *S. hominis* is shorter than *S. chromogenes* or *S. simulan*.

Superinfection with *S. hominis* is associated with pronounced fever and clinical manifestations, particularly in cows with a pre-existing infection (COW1, COW2, and COW4) that were previously infected with *S. aureus*, *S. uberis*, or *S. agalactiae*. The persistence of these bacteria is already inducing tissue damage and the accumulation of inflammatory cells in the udder, resulting in a primed inflammatory state [16,25,26]. Although *S. hominis* may have already been eliminated from the tissue, peptidoglycan or lipoteichoic acid, as components of the remaining bacterial cell wall, could continue to stimulate an innate immune response, leading to sustained production of inflammatory mediators and thereby contributing to more pronounced clinical signs or elevated body temperature [27]. In contrast, *S. hominis* establishment is minimal, and clinical symptoms are significantly less pronounced in uninfected cows (COW3). This observation is consistent with the general behavior of coagulase-negative staphylococci, which tend to induce mild, short-lived, or subclinical intramammary infections (IMI) that are less persistent than those caused by major pathogens [28].

The clinical manifestation depends on the bacterial load in the udder. A higher bacterial load leads to an increased pathogen-associated molecular pattern (PAMP) response, which stimulates the release of cytokines and other inflammatory mediators, resulting in more severe clinical manifestations [29]. COW1 exhibited the most severe mastitis, with multiple infections, primarily due to *S. aureus*, and superinfections with Gram-negative bacteria in those quarters. Regarding *S. aureus’s* ability to establish chronic infection through intracellular invasion, biofilm formation, and evasion of immune response, this phenomenon is associated with elevated somatic cell count and continuous shedding of a large number of bacteria into quarter milk, resulting in a higher bacterial count that remains above the infection threshold [30,31]. In addition, lipopolysaccharide (LPS) of Gram-negative bacteria activates a strong immune response by binding to the toll-like receptor 4 (TLR4) and stimulating the expression of chemokines (such as IL-8) and pro-inflammatory cytokines (such as IL-6 and IL-1β) [32]. Superinfection under such pre-existing infection conditions may act as an inflammatory amplifier, leading to more severe clinical symptoms, especially at high bacterial loads. An unexpected finding was that the bacterial count of SH-IMI at a concentration of 10^5^ was the highest and significantly greater than those at concentrations of 0 and 10^6^. The 10^5^ CFU/mL concentration was the only concentration administered to both COW1 and COW3, whereas the 10^6^ CFU/mL concentration was administered to COW1 and COW2. Therefore, the higher bacterial counts observed at 10^5^ CFU/mL may reflect differences between cows and their baseline infection status rather than an intrinsic non-linear effect of inoculum concentration. In addition, the cow and housing site might affect the results.

In this study, the uncountability SCC from clinical mastitis quarters was adjusted to 9999 × 10^3^ cells/ml, as the highest value from somatic cell counters. In general, SCC reflects a high udder immune response, causing more severe mastitis. However, replacing the high number as in this study might be a problematic imputation method that could have caused heavily inflated group means and distorted standard errors, even after logarithm-transforming the data. The application of SCC results must be performed carefully. Fundamental variations in bacterial inflammatory mechanisms are reflected in the response of somatic cell levels (SCC) to different bacterial infections identified in this study. SCC levels align with the bacterial count findings. The consistently elevated somatic cell count and bacterial counts across all quarters of COW1 indicate chronic inflammation due to a persistent *S. aureus* infection. The characteristics of *S. aureus* infection include the ability to evade the host immune response and to form clusters (biofilms), which can induce chronic inflammation and create gaps in mammary gland tissue [26]. The ability of *S. aureus* to express various superantigens and produce toxins such as α-hemolysin and γ-hemolysin contributes to the lysis of somatic and white blood cells. Additionally, *S. aureus* has peptidoglycan and lipoteichoic acid, which are pathogen-associated molecular patterns (PAMPs) recognized by toll-like receptors (TLRs) on the surfaces of mammary epithelial cells and local immune cells [33]. The recognition of these PAMPs leads to prolonged inflammation due to the production of pro-inflammatory cytokines such as TNF-α, IL-1β, and IL-6, resulting in the continuous recruitment of white blood cells into the mammary tissue. The somatic cell counts in two quarters of COW2 consistently exceeded the subclinical mastitis threshold throughout the study, indicating persistent mammary inflammation due to *S. uberis*. Consistent with the findings of [34], *S. uberis* can infect mammary tissue and spread infection due to its production of hyaluronidase and peptidase, two key virulence factors that cause severe inflammation [34]. In addition, *S. uberis* can induce a persistent inflammatory response characterized by the production of high amounts of interleukin-6 (IL-6) and interleukin-8 (IL-8) in milk and mammary gland tissues [34]. In contrast, SCC in COW3 was temporarily increased following SH-IMI, reaching subclinical mastitis levels, suggesting that *S. hominis* elicits a lower inflammatory response than major mastitis pathogens. The study by Taponen and Pyörälä [11] supported that *S. hominis* is a low-inflammatory pathogen that often resolves on its own, causing only minor damage to the mammary gland tissue. This somatic cell response in cows indicates that the local immune system of the mammary gland can effectively control this infection. In COW4, the SCC appears to be influenced primarily by the pre-existing *S. agalactiae* infection (Q1 and Q3). The characteristics of *S. agalactiae* infections that cause subclinical mastitis and exhibit relatively lower somatic cell counts than those of *S. aureus* and *S. uberis* are associated with specific immune evasion mechanisms. *S. agalactiae* possesses a sialic acid capsular polysaccharide, which allows the bacteria to mimic the host cell surface and effectively evade recognition by the complement system [35]. Additionally, *S. agalactiae* can produce superoxide dismutase and C5a peptidase, which reduce the recruitment of white blood cells into the mammary tissue [36]. This results in a milder inflammatory response, primarily involving the recruitment of the innate immune system through macrophages rather than inducing a severe adaptive immune response [37]. The balance between the ability to persist and to avoid excessive inflammation allows *S. agalactiae* to establish chronic infections with moderate SCC without causing severe inflammation, as in *S. aureus* or *S. uberis* infections.

MDA is a primary biomarker for oxidative stress, indicating cellular damage [38]. The highest average MDA concentrations were observed in COW1, which was chronically infected with *S. aureus*, indicating persistent oxidative stress and more extensive, irreversible mammary tissue injury compared with the other cows. This condition may have resulted from the chronic inflammatory state induced by pre-existing *S. aureus* infection rather than from the experimental SH-IMI alone. Similarly, *S. uberis*-infected quarters of COW2 also showed high MDA concentrations, indicating progressive damage of mammary epithelial tissue from ongoing leukocyte infiltration and chronic inflammation [39]. Interleukin-6 (IL-6) is a major pro-inflammatory cytokine and an early biomarker of udder infection, while interleukin-4 (IL-4) is a regulatory cytokine that modulates the immune response and promotes tissue repair [15,40]. COW1 and COW2 exhibited elevated levels of IL-4 and IL-6, indicating that inflammatory and regulatory pathways occur simultaneously during intense bacterial infection. The average of IL-6 in three-quarters of COW1 was the lowest. This phenomenon can be explained by immune tolerance, which impairs leukocyte function or downregulates inflammatory signaling pathways, thereby affecting the release of proinflammatory mediators in chronic conditions [41]. IL-4 levels across all quarters of each cow were similar, with IL-4 in COW2 the highest, followed by COW1, COW4, and COW3, suggesting that variation in immune regulation is individual-specific rather than a direct effect on SH-IMI. This is consistent with the previous report demonstrating that IL-4 responses exhibit greater stability (i.e., are less dynamic) than those of pro-inflammatory cytokines such as IL-6 [42].

The levels of IL-4 and IL-6 were associated with the period of SH-IMI, with levels on days 1–3 significantly higher than before. This supports the roles of these biomarkers in the response after bacterial infection. In contrast, these parameters were significantly influenced by both individual cow factors and the SH-IMI challenge concentration, indicating that oxidative stress depends not only on the experimental infection but also on the underlying physiological and infection status of individual animals. The unexpectedly higher MDA concentration in the 10^5^ CFU SH-IMI group compared with the 10^6^ CFU group may be attributable to individual cow effects, differences in bacterial establishment, or variation in host immune responses, rather than to a simple dose-dependent relationship. Similar non-linear relationships between bacterial dose and inflammatory markers have been reported in experimental mastitis models, where moderate bacterial burdens may establish infection more successfully while avoiding immediate elimination by a strong immune response.

The results from malondialdehyde (MDA) and cytokine analyses indicate that *S. hominis* intramammary infection (SH-IMI) induced a transient, acute inflammatory response characterized by short-term increases in IL-6 levels. In contrast, MDA levels reflected longer-term oxidative stress and tissue damage, which were strongly influenced by the pre-existing infection status of individual cows. The consistently elevated MDA concentrations and suppressed IL-6 responses in COW1 support the hypothesis that chronic *S. aureus* infection altered mammary immune function and contributed to persistent oxidative injury. Consequently, the significantly higher MDA concentrations observed in COW1 and COW2 are likely attributable to the profound confounding effects of pre-existing major pathogens (*S. aureus* and *S. uberis*), rather than the SH-IMI dose alone. Conversely, the minimal oxidative stress in COW3 and COW4 indicates that high bacterial loads of *S. hominis* do not independently cause severe mammary tissue damage. Thus, the individual cow factor, specifically their pre-existing infection status, was the primary driver of the observed differences in MDA and IL-6 concentrations. Meanwhile, IL-4 levels remained relatively stable throughout, indicating a limited role in differentiating the inflammatory responses observed among the cows in this study.

The limitation of this study was the small number of cows enrolled; all animals had pre-existing intramammary infections and a history of antibiotic and anti-inflammatory treatments. This study primarily serves as an in vivo proof-of-concept to elucidate the mechanisms of bacterial interference and innate immune priming, was exploratory, and was not designed or intended to establish a dose–response relationship. In addition, the current study relies on a single pre-challenge baseline measurement per quarter for MDA, IL-4, and IL-6. This single observation does not account for the substantial within-quarter day-to-day variability typical of milk cytokines and oxidative stress markers, which should be considered when interpreting the magnitude of the post-challenge responses. The unequal distribution of inoculum concentrations among cows, quarter milk yield, milk compositions, together with differences in pre-existing intramammary infections and housing sites, resulted in potential confounding between inoculum concentration and cow-level characteristics. These factors may have influenced the outcomes and potentially confounded the relationship between *S. hominis* intramammary infection (SH-IMI), mammary tissue recovery, and persistence of infection. Further studies should include a larger number of animals and better-controlled experimental conditions. For example, cows with similar pre-existing infections, comparable infecting bacterial strains, and standardized treatment histories should be selected to minimize variability and allow for a more accurate assessment of inflammatory responses. More frequent sampling during the first 24 h post-inoculation should also be considered, as *S. hominis* appears to persist only briefly in the udder, and important early inflammatory changes may have been missed. In addition, using multiple *S. hominis* strains would provide a more comprehensive understanding of strain-dependent differences in inflammatory response dynamics. Molecular approaches such as whole-genome sequencing or strain typing should be employed to distinguish true superinfections caused by newly introduced microorganisms from the proliferation of pre-existing bacterial populations present at low abundance. Such analyses would improve our understanding of microbial interactions within the mammary gland and help clarify their relationship with infection persistence, host inflammatory responses, and clinical outcomes. Future translational research aiming for on-farm application must focus on isolating the specific bioactive components of *S. hominis*, such as purified bacteriocins or postbiotic immunomodulators, to develop safe, non-living therapeutic formulations.

## 5. Conclusions

SH-IMI was associated with increased body temperature and clinical signs, although the degree of response varied among cows and periods. High-dose SH-IMI exposure intensifies immune stimulation, leading to increased cytokine production and oxidative stress in the mammary gland. Although *S. hominis* is often considered less pathogenic than major mastitis pathogens, the present results indicate that high bacterial loads may still induce substantial inflammatory and oxidative responses, potentially contributing to mammary tissue damage and clinical manifestations of mastitis.

## Figures and Tables

**Table 1:** Cows and study design and results of superimposed experimental intramammary infection (IMI) with *Staphylococcus hominis* (SH) using cows with historically chronic mastitis.

COW	Quarter	SH-IMI ^3^	SH-D1	Fever-D1	CM-D1	IMI-D0	IMI-D1	IMI-D10
Farm A ^1^								
COW1	Left Fore	10^6^	No	Yes	Yes	AU	AU	AU
	Right Fore	10^6^	No		No	AU	AU	AU
	Left Right	10^5^	No		Yes	AU	AU	AU
	Right Right	10^5^	No		Yes	AU	AU	AU
COW2	Left Fore	0	No	Yes	No	-	-	-
	Right Fore	10^6^	No		Yes	-	AU	-
	Left Right	10^6^	No		Yes	SU	SU	-
Training university barn ^2^								
COW3	Left Fore	10^4^	Yes	No	No	-	SA	-
	Right Fore	10^4^	Yes		No	-	-	-
	Left Right	10^5^	Yes		Yes	-	-	-
	Right Right	10^5^	Yes		No	-	-	-
COW4	Left Fore	10^4^	No	Yes	Yes	SA	SA	SA
	Right Fore	0	No		No	SCM	SCM	-
	Left Right	10^4^	No		Yes	SA	SA	SA

^1^ Farm A used for study in Nov. 2024 with health monitoring and treatment by farmers and a local veterinarian, respectively. ^2^ Training university barn used for study in Feb 2025 with health monitoring and treatment by veterinary students and staff, Faculty of Veterinary Medicine, Chiang Mai University. ^3^ SH-IMI indicating concentrations of SH for experimental IMI with SH. Abbreviations: F (Front), H (Hind), AU (*Staphylococcus aureus*), SU (*S. uberis*), SCM (*S. chromogenes*), SA (*Streptococcus agalactiae*), IMI (intramammary infection).

## Data Availability

The raw dataset and model estimates supporting the findings of this study are available upon request from the corresponding author.

## Abbreviations

The following abbreviations are used in this manuscript:

SH	*Staphylococcus hominis*
SH-IMI	Intramammary infection with *S*. *hominis*
SCC	Somatic cell count
NAS	Non-aureus staphylococci
IMI	Intramammary infection
F	Front
H	Hind
AU	*Staphylococcus aureus*
NPU	Non-producing unit
SCM	Subclinical mastitis
SA	*Streptococcus agalactiae*
LF	Left-front
RF	Right-front
LH	Left->hind
RH	Right-hind
MDA	Malondialdehyde
pi	post infection
